# Sustainable Route for Synthesizing Aluminosilicate EU-1 Zeolite

**DOI:** 10.3390/molecules26051462

**Published:** 2021-03-08

**Authors:** Hao Xu, Jie Zhu, Xiong Wang, Chao Shen, Shengshen Meng, Kai Zheng, Chao Lei, Longfeng Zhu

**Affiliations:** 1 College of Biology and Environmental Engineering, Zhejiang Shuren University, Hangzhou 310015, China; xuhao@zju.edu.cn (H.X.); shenchaozju@zjsru.edu.cn (C.S.); zkai86@163.com (K.Z.); chaolei_212@163.com (C.L.); 2 College of Biological, Chemical Science and Engineering, Jiaxing University, Jiaxing 314001, China; zhuyaojie@zjxu.edu.cn (J.Z.); 00171670@zjxu.edu.cn (S.M.); 3 Ningbo Rail Transit, Ningbo 315101, China; wangxiong@nbmetro.com

**Keywords:** zeolite, aluminosilicate EU-1, sustainable route, solvent-free

## Abstract

Developing sustainable routes for the synthesis of zeolites is still a vital and challenging task in zeolite scientific community. One of the typical examples is sustainable synthesis of aluminosilicate EU-1 zeolite, which is not very efficient and environmental-unfriendly under hydrothermal condition due to the use of a large amount of water as solvent. Herein, we report a sustainable synthesis route for aluminosilicate EU-1 zeolite without the use of solvent for the first time. The physicochemical properties of the obtained EU-1 zeolite are characterized by powder X-ray diffraction (XRD), scanning electron microscopy (SEM), thermogravimetry-differential thermal analysis (TG-DTA), N_2_ sorption, inductively coupled plasma (ICP) analysis, and solid nuclear magnetic resonance (NMR), which show the product has high crystallinity, uniform morphology, large BET surface area, and four-coordinated aluminum species. Moreover, the impact of synthesis conditions is investigated in detail. The sustainable synthesis of aluminosilicate EU-1 zeolite under solvent-free

## 1. Introduction

Zeolites are crystalline microporous materials, which have widespread applications in diverse areas, such as ion-exchanging, gas adsorption, and shape-selective catalysis [1,2,3,4,5,6]. The common methods for preparing zeolites including hydrothermal, solvothermal, and ionothermal synthesis routes normally require the presence of different kinds of solvents. The use of solvents always leads to a lot of problems, such as high autogeneous pressure during the synthesis process, low utilization efficiency of the autoclaves, low solid yields, and pollution from waste solvents [7,8,9,10,11]. In the past decade, Xiao’s group has reported a sustainable solvent-free synthesis route for many kinds of aluminosilicate zeolites and aluminophosphates-based zeolites, which successfully solve the aforementioned problems [12,13,14,15,16].

EU-1 zeolite possesses a one-dimensional channel system of 10-membered-ring channels (0.58 × 0.41 nm) running along the [100] direction, which is connected with 12-membered-ring side pockets (0.68 × 0.58 × 0.80 nm) in the [001] direction. Due to this unique structure, aluminosilicate EU-1 zeolite shows superior catalytic performances in a series of catalytic reactions especially in *m*-xylene isomerization reaction [17,18,19,20,21,22,23,24,25,26,27,28]. Therefore, the research on synthesis methods of aluminosilicate EU-1 zeolite is of significance. Normally, aluminosilicate EU-1 zeolite is prepared under hydrothermal condition in the presence of organic templates or zeolite seeds [27,28,29]. The preparation of EU-1 zeolite using hydrothermal method usually has disadvantages of low solid yield, waste water pollution and so on. In addition, the dry-gel conversion method was used for the preparation of aluminosilicate EU-1 zeolite. In dry-gel conversion process, a prepared damp or dried sodium aluminosilicate gel suspended above a liquid in a sealed autoclave was subjected to a mixed vapor of water at elevated temperature and pressure. It is worth noting that the large amounts of solvents, such as water, are necessary in the preparation of damp or dried sodium aluminosilicate gel [25,30]. Currently, the solvent-free synthesis method of pure silica EUO zeolite was successfully achieved by Wu et al. in the presence of fluoride species [31]. However, the fluoride species are highly toxic, which is still not sustainable from the viewpoint of green chemistry. Now, the true sustainable synthesis of aluminosilicate EU-1 zeolite without the use of fluoride species is not successful yet.

Herein, we report, for the first time, a successful synthesis of aluminosilicate EU-1 zeolite using the sustainable solvent-free route. Notably, the solid yield of the aluminosilicate EU-1 zeolite product is as high as 99%, which is very outstanding compared with that of the hydrothermal synthesis (88%) [20].

## 2. Results and Discussion

**Figure 1:**
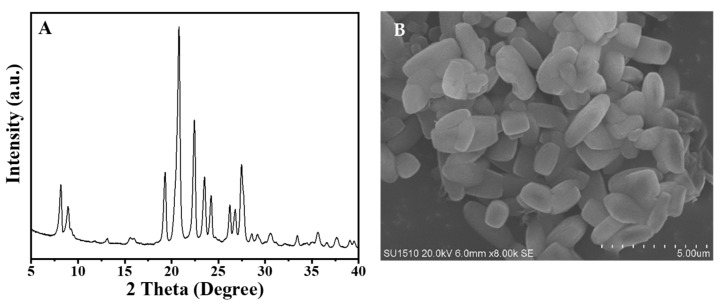
(**A**) XRD pattern and (**B**) SEM image of the S-EU-1 zeolite, respectively.

**Figure 2:**
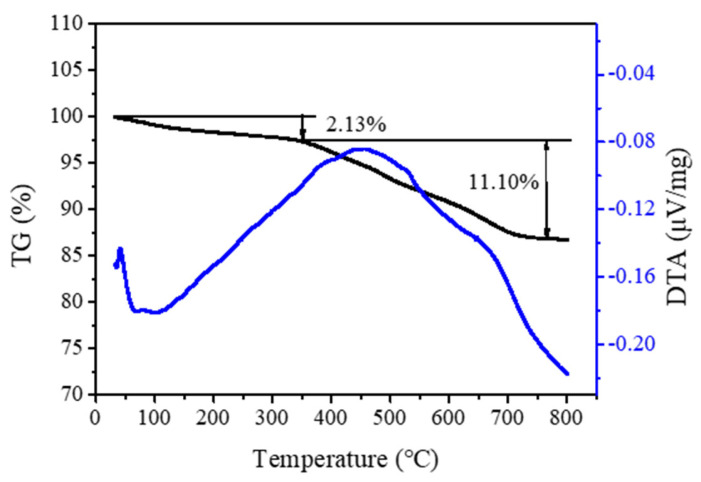
Thermogravimetry-differential thermal analysis (TG-DTA) curves of the S-EU-1 zeolite.

**Figure 3:**
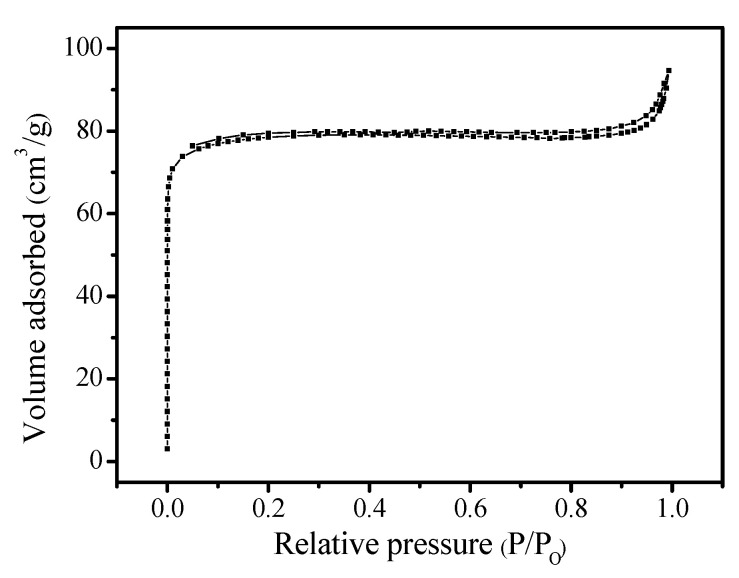
N_2_ sorption isotherm of the H-S-EU-1 zeolite.

Figure 1A shows the XRD pattern of the S-EU-1 zeolite, which displays the similar peaks (8.16, 8.94, 19.3, 20.7, and 22.4°) with that of the simulated XRD pattern of EU-1 zeolite from IZA (Figure S1), as well as the XRD pattern of the C-EU-1 zeolite (Figure S2) [20]. This result shows that the high crystallinity of EU-1 zeolite would indeed be obtained. Figure 1B gives the SEM image of the S-EU-1 zeolite, showing the uniform spheroidicity morphology, which is very similar with that of the C-EU-1 zeolite (Figure S3). This result suggests the high quality of EU-1 zeolite with perfect morphology could be obtained, in good agreement with the result of XRD pattern. Figure 2 shows the thermal analysis (thermogravimetry-differential thermal analysis (TG-DTA)) of the S-EU-1 zeolite. In the TG-DTA curves, it exhibits the major exothermic peaks at 200–800 °C accompanied by the weight loss at about 11.1% associated with the decomposition of organic structure directing agent in the micropores of the S-EU-1 zeolite. Figure 3 gives the N_2_ sorption isotherm of the H-S-EU-1 zeolite. A steep adsorption increase occurs in the relative pressure (10^−6^ < P/P_0_ < 0.01), which is due to the filling of zeolite micropores by N_2_. Correspondingly, the micropore volume and the BET surface area of the H-S-EU-1 zeolite are measured at about 0.11 cm^3^/g and 264 m^2^/g, respectively, calculated by the t-plot and BET methods, which are comparable to that of the conventional aluminosilicate EU-1 zeolite reported in the literatures [20,22,32]. Moreover, it is worth mentioning that the solid yield of the H-S-EU-1 zeolite is as high as 99% due to the avoidance of the aluminosilicate dissolution in the absence of water solvent.

**Figure 4:**
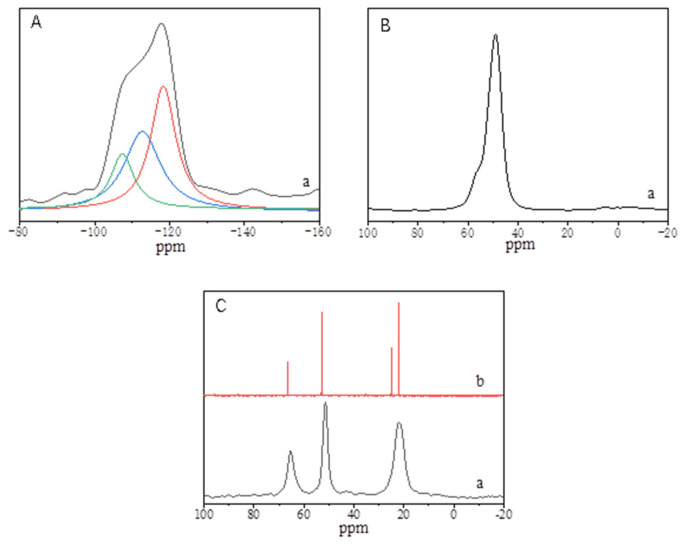
(**A**) ^29^Si, (**B**) ^27^Al, and (**C**) ^13^C solid nuclear magnetic resonance (NMR) spectra of the (a) S-EU-1 zeolite and (b) ^13^C liquid NMR spectrum of hexamethonium bromide (HMBr_2_) in D_2_O solution, respectively.

Figure 4 shows the ^29^Si, ^27^Al, and ^13^C MAS NMR spectra of the S-EU-1 zeolite. Figure 4A shows the solid ^29^Si MAS NMR spectrum of the S-EU-1 zeolite, giving peaks at about −118.3 ppm, −112.7 ppm and −107.4 ppm. The peaks at −118.3 and −112.7 ppm are assigned to Si (4Si, 0Al) species, while the peak at −107.4 ppm is assigned to Si (3Si, 1Al) and/or Si (3Si, 1OH) species [20,30,33]. Very interestingly, the area proportion of the peak at −107.4 ppm is about 17.5%, which suggests the SiO_2_/Al_2_O_3_ ratio of the S-EU-1 zeolite is about 45.7. This result is in good agreement with the SiO_2_/Al_2_O_3_ ratio (45.2) tested by ICP-OES technique. The solid ^27^Al-NMR spectrum of the S-EU-1 zeolite in Figure 4B gives the peaks at 40–60 ppm, which are assigned to 4-coordinated aluminum species in the zeolite framework. In addition, the absence of the signal around 0 ppm shows that there is no 6-coordinated aluminum species in the S-EU-1 zeolite. This result suggests that it is indeed obtained the aluminosilicate EU-1 zeolite with good aluminum species coordination. Figure 4C shows the solid ^13^C-NMR spectrum of the S-EU-1 zeolite and the liquid ^13^C-NMR spectrum of the organic template of HMBr_2_ molecules. The peaks in the two spectra are very consistent, showing that the HMBr_2_ molecules are indeed located in the channel of the S-EU-1 zeolite.

**Figure 5:**
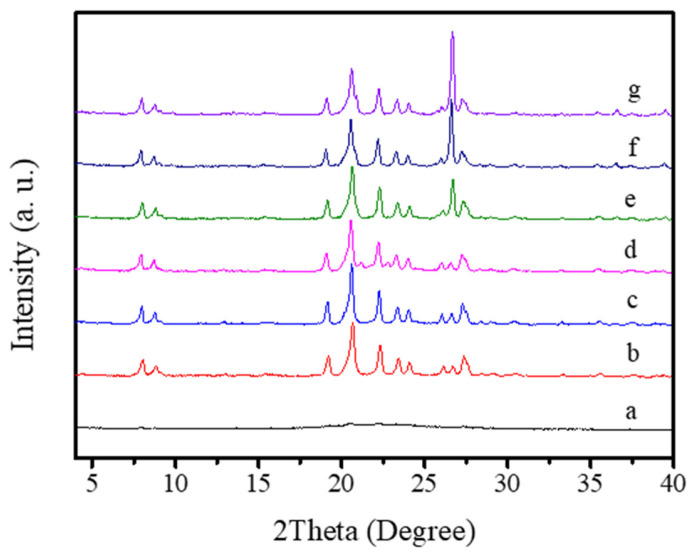
XRD patterns of the samples with different SiO_2_/Al_2_O_3_ ratios (a: SiO_2_/Al_2_O_3_ = 30, b: SiO_2_/Al_2_O_3_ = 50, c: SiO_2_/Al_2_O_3_ = 60, d: SiO_2_/Al_2_O_3_ = 70, e: SiO_2_/Al_2_O_3_ = 80, f: SiO_2_/Al_2_O_3_ = 100, and g: SiO_2_/Al_2_O_3_ = 120).

**Figure 6:**
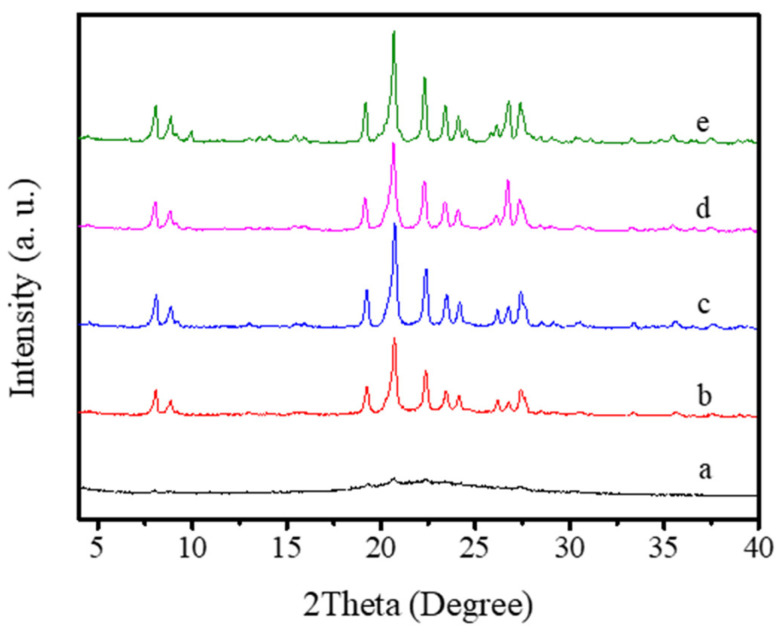
XRD patterns of the samples with different Na_2_O/SiO_2_ ratios (a: Na_2_O/SiO_2_ = 0.080, b: Na_2_O/SiO_2_ = 0.10, c: Na_2_O/SiO_2_ = 0.12, d: Na_2_O/SiO_2_ = 0.14, e: Na_2_O/SiO_2_ = 0.16).

**Figure 7:**
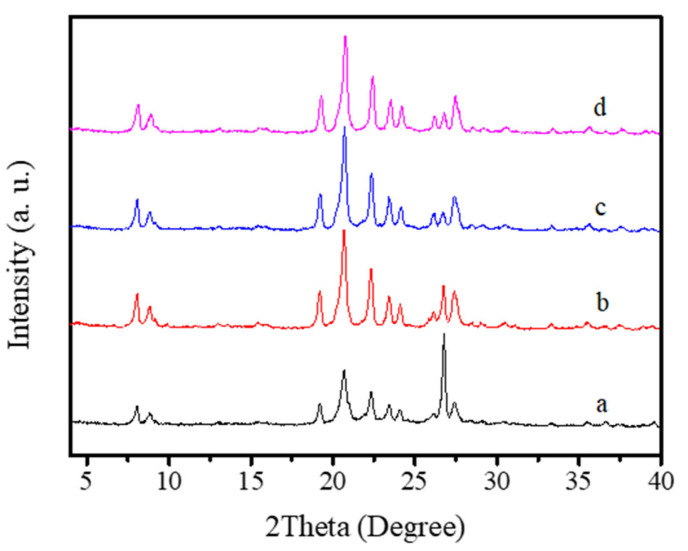
XRD patterns of the samples with different HMBr_2_/SiO_2_ ratios (a: HMBr_2_/SiO_2_ = 0.010, b: HMBr_2_/SiO_2_ = 0.30, c: HMBr_2_/SiO_2_ = 0.042, d: HMBr_2_/SiO_2_ = 0.080).

**Figure 8:**
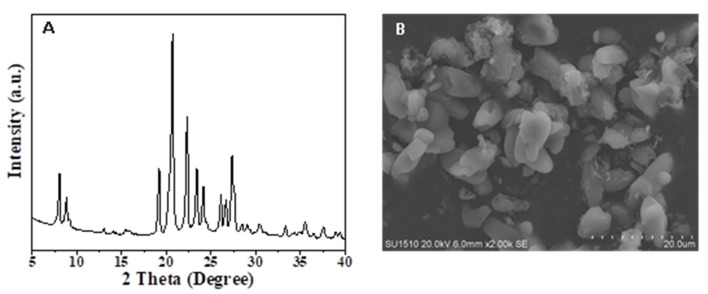
(**A**) XRD pattern and (**B**) SEM image of the sample synthesized without addition of the EU-1 zeolite seeds, respectively.

Table 1 and Figure 5, Figure 6, Figure 7 and Figure 8 present the effects of synthesis conditions on the crystallization of the S-EU-1 zeolite. When the SiO_2_/Al_2_O_3_ ratio in the starting solid mixture is about 30, the product remains amorphous (Run 1, Table 1; Figure 5a); varying the SiO_2_/Al_2_O_3_ ratio in the starting solid mixture from 50 to 70, all of the products are pure EU-1 zeolites (Run 2–4, Table 1; Figure 5b,d); When the SiO_2_/Al_2_O_3_ ratio in the starting solid mixture is higher than 80, the products contain EU-1 zeolite and dense phase (Run 5–7, Table 1; Figure 5e,g).

In addition, the Na_2_O/SiO_2_ ratio in the starting solid mixture is very vital. It is found that the relatively low Na_2_O/SiO_2_ ratios (0.080–0.10) in the starting solid mixture would result in incomplete crystallization of EU-1 zeolite (Run 8–9, Table 1; Figure 6a,b). When the Na_2_O/SiO_2_ ratio in the starting solid mixture grows to 0.12, the product is pure EU-1 zeolite with good crystallinity (Figure 6c). Further increasing the Na_2_O/SiO_2_ ratio in the starting solid mixture from 0.14 to 0.16, the products become the mixture of EU-1 zeolite and dense phase (Run 10–11, Table 1; Figure 6d,e).

Furthermore, the ratios of HMBr_2_/SiO_2_ in the synthesis is carefully studied. The relatively low HMBr_2_/SiO_2_ ratios (0.010–0.030) would lead to the growth of dense phase with EU-1 zeolite (Run 12–13, Table 1; Figure 7a,b). While the relatively high HMBr_2_/SiO_2_ ratio (0.080) has no effect in the crystallization of EU-1 zeolite (Run 14, Table 1; Figure 7c,d).

Moreover, when the EU-1 zeolite seed is absent, the product is still pure phase EU-1 zeolite (Run 15, Table 1) with high crystallinity, which is shown in Figure 8A. However, the SEM image of EU-1 zeolite in Figure 8B shows that the morphology of the aforementioned product is not as perfect as the EU-1 zeolite synthesized with the addition of EU-1 zeolite seeds, which could clearly observe the small amount of amorphous phase that could not be observed from the XRD pattern.

**Figure 9:**
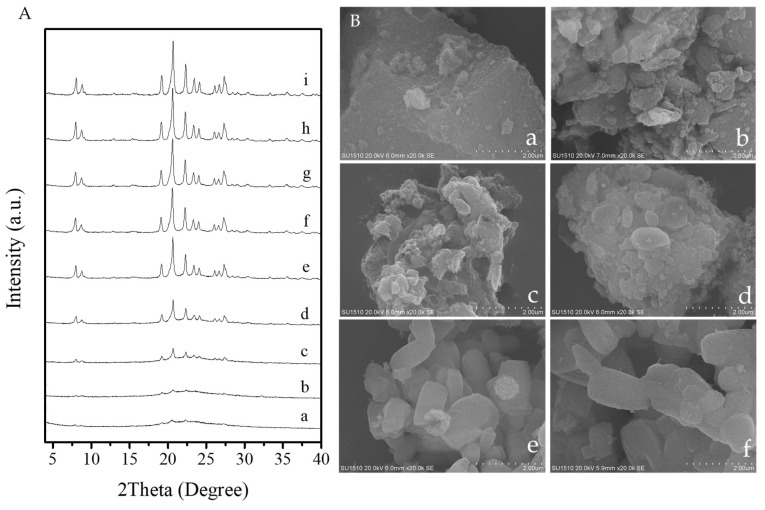
(**A**) XRD patterns of the S-EU-1 zeolite crystallized at (**a**) 0, (**b**) 3, (**c**) 6, (**d**) 9, (**e**) 12, (**f**) 18, (**g**) 24, (**h**) 36, (**i**) 48 h, respectively. (**B**) SEM images of the S-EU-1 zeolite crystallized at (**a**) 0, (**b**) 3, (**c**) 6, (**d**) 9, (**e**) 24, (**f**) 48 h, respectively.

**Figure 10:**
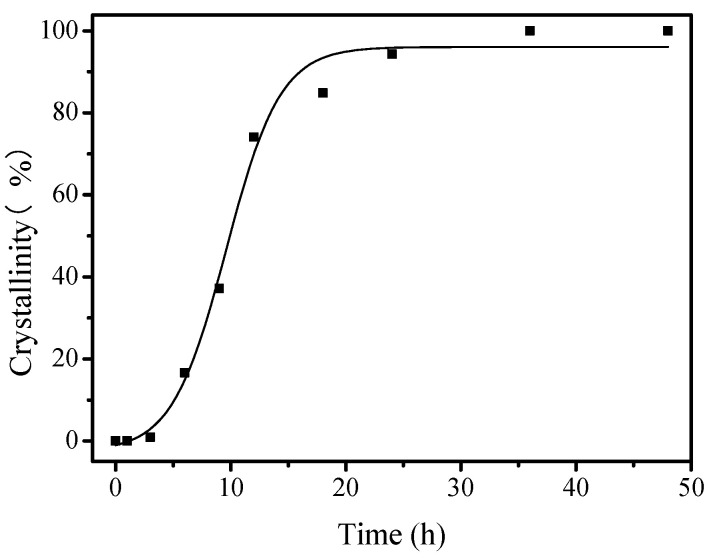
The dependence of the S-EU-1 zeolite crystallinity on crystallization time.

Figure 9 shows the synthesis process of the S-EU-1 zeolite monitored by XRD and SEM techniques. Before crystallization, the XRD pattern shows a series of weak peaks associated with EUO topology, which is associated with the EU-1 zeolite seeds added in the synthesis system (Figure 9A(a)). The SEM image of the sample before crystallization is basically amorphous (Figure 9B(a)). Increasing the crystallization time from 3 to 6 h, the intensities of the XRD peaks assigned to EU-1 zeolite became stronger (Figure 9A(b,c)). Correspondingly, a small amount of zeolite crystals could be observed via the SEM images (Figure 9B(b,c)). Further increasing the crystallization time from 9 to 36 h, the intensities of XRD peaks continue to rise (Figure 9A(d,h)). At the same time, more ellipsoidal crystals of the S-EU-1 zeolite would be found in the products (Figure 9B(d) and Figure 9B(e)). When the crystallization time reaches to 48 h, there was no obvious changes on peak intensity observed in the XRD pattern (Figure 9A(i)), suggesting the complete crystallization of the EU-1 zeolite. Moreover, the SEM image (Figure 9B(f)) shows the perfect EU-1 zeolite crystals after crystallized for 48 h. Figure 10 gives the dependence of the crystallinity of S-EU-1 zeolite on crystallization time. Compared with the conventional synthesis, this sustainable route has successfully reduced the crystallization time of aluminosilicate EU-1 zeolite.

## 3. Materials and Methods

### 3.1. Starting Materials

Sodium metasilicate nonahydrate (NaSiO_3_•9H_2_O, AR, Na_2_O of 19.3~22.8 wt%, Sinopharm Chemical Reagent Co., Ltd., Shanghai, China), aluminum sulfate octadecahydrate (Al_2_(SO_4_)_3_•18H_2_O, AR, 99%, Aladdin Chemical Co., Ltd., Shanghai, China), solid silica gel (Qingdao Haiyang Chemical Reagent Co., Ltd., Qingdao, China), sodium meta-aluminate (NaAlO_2_, AR, 99%, Sinopharm Chemical Reagent Co., Ltd., Shanghai, China), sodium hydroxide (NaOH, AR, 96%, Sinopharm Chemical Reagent Co., Ltd., Shanghai, China), colloidal silica (40 wt% SiO_2_ in water, Sigma-Aldrich Reagent Co., Ltd., Shanghai, China), hexamethonium bromide (HMBr_2_, 98%, J&K Scientific Ltd., Beijing, China), and ammonium nitrate (NH_4_NO_3_, AR, 99%, Beijing Chemical Reagent Co., Ltd., Beijing, China) are used without further purification. The deionized water was produced by a deionized water system from ULUPURE UPT-Ⅰ with a resistivity of 17.0–8.2 Mohm.cm.

### 3.2. Hydrothermal Synthesis of Conventional Aluminosilicate EU-1 Zeolite

In a typical run for synthesizing conventional aluminosilicate EU-1 zeolite under hydrothermal condition, 0.066 g of sodium meta-aluminate and 0.18 g of sodium hydroxide was dissolved into 13.55 g deionized water. Then, 0.95 g of hexamethonium bromide was added into the mixture. After stirring for 30 min, 2.5 g of colloidal silica was added dropwise. Finally, the mixtures were stirred for 2 h and transferred into a Teflon-lined autoclave oven, sealed, and crystallized at 180 °C for 72 h under rotation condition (50 rpm). After filtering, washing, and drying, the product was obtained (designated as C-EU-1) [20,22].

### 3.3. Solvent-Free Synthesis of Aluminosilicate EU-1 Zeolite

In a typical run for synthesizing aluminosilicate EU-1 zeolite without addition of water as solvent, 2.81 g of SiO_2_•0.5H_2_O (hydrated form of solid silica gel), 1.11 g of sodium metasilicate nonahydrate, 0.37 g of aluminum sulfate octadecahydrate, 0.5 g of hexamethonium bromide, and 0.02 g of EU-1 zeolite seeds (H-C-EU-1) were added together. After grinding for 5–10 min, the powder mixtures were transferred to a Teflon-lined autoclave oven, sealed and crystallized at 180 °C for 48 h under static condition. After filtering, washing, and drying, the product was obtained (designated as S-EU-1). The H-form of the sample (designated as H-S-EU-1) was obtained by calcining the S-EU-1 zeolite at 550 °C for 4 h in the air atmosphere, ion-exchanging with 1M NH_4_NO_3_ solution twice at 80 °C for 2 h, and then calcining at 500 °C for 4 h in the air atmosphere.

### 3.4. Methods

X-ray powder diffraction (XRD) patterns were measured with a Rigaku Ultimate VI X-ray diffractometer (Tokyo, Japan, 40 kV, 40 mA) using Cu_Kα_ (λ = 1.5406 Å) radiation. Scanning electron microscopy (SEM) experiments were performed on Hitachi SU-1510 electron microscopes (Tokyo, Japan). The samples were covered with gold. The N_2_ sorption isotherms at the temperature of liquid nitrogen (−196 °C) were measured using Micromeritics ASAP 2020M (Micromeritics Instrument Corporation, Atlanta, GA, USA) and Tristar system. The samples were outgassed for 10 h at 200 °C before the measurements. The pore volume and surface area were calculated from the t-plot and BET methods. The thermogravimetric analysis (TGA) experiments were carried out on a Perkin-Elmer TGA 7 (Waltham, MA, USA). Here, 10 mg of the sample was subjected to an air flow rate of 120.0 mL/min, and the test was programmed at a heating rate of 10 °C/min, in the temperature range from room temperature to 800 °C. Solid-state ^29^Si, ^27^Al and ^13^C MAS nuclear magnetic resonance (NMR) spectra were recorded on an Agilent 600M spectrometer (Santa Clara, CA, USA). Liquid ^13^C-NMR spectrum was recorded on a Bruker Avance 500 spectrometer (Leipzig, Germany). The sample composition was determined by inductively coupled plasma (ICP) with a Perkin-Elmer 3300DV emission spectrometer (Waltham, MA, USA).

## 4. Conclusions

In summary, we developed a sustainable synthesis route for preparing aluminosilicate EU-1 zeolite. The advantages accompanied by the avoidance of the use of solvent include high solid yield, low autogeneous pressure during the synthesis process, high space utilization of the autoclave, and low waste water pollution. The obtained aluminosilicate EU-1 zeolite shows high crystallinity, uniform morphology, high BET surface area, and good aluminum species coordination. The aforementioned properties show the high quality of the obtained product and thus indicate the good application prospect of the aluminosilicate EU-1 zeolite synthesized from solvent-free method. We believe that this sustainable synthesis method might offer an opportunity for the industrial applications of aluminosilicate EU-1 zeolite in the near future.

## Figures and Tables

**Table 1:** Sustainable synthesis of aluminosilicate EU-1 zeolite under various conditions.

Run ^1^	SiO_2_/Al_2_O_3_	Na_2_O/SiO_2_	HMBr_2_/SiO_2_	Seeds/SiO_2_	Products ^2^
1	30	0.12	0.042	0.010	Amorphous
2	50	0.12	0.042	0.010	EU-1
3	60	0.12	0.042	0.010	EU-1
4	70	0.12	0.042	0.010	EU-1
5	80	0.12	0.042	0.010	EU-1 + dense phase
6	100	0.12	0.042	0.010	EU-1 + dense phase
7	120	0.12	0.042	0.010	EU-1 + dense phase
8	60	0.080	0.042	0.010	Amorphous
9	60	0.10	0.042	0.010	EU-1 + Amorphous
10	60	0.14	0.042	0.010	EU-1 + dense phase
11	60	0.16	0.042	0.010	EU-1 + dense phase
12	60	0.12	0.010	0.010	EU-1 + dense phase
13	60	0.12	0.030	0.010	EU-1 + dense phase
14	60	0.12	0.080	0.010	EU-1
15	60	0.12	0.042	0	EU-1

^1^ The samples are synthesized at 180 °C for 48 h. ^2^ The phase appearing first is dominant.

## Data Availability

The data presented in this study are available on request from the corresponding author.

## Supplementary Materials

The following are available online, Figure S1: Simulated XRD pattern of the EU-1 zeolite, Figure S2: XRD pattern of the C-EU-1 zeolite, and Figure S3: SEM image of the C-EU-1 zeolite.

## Sample Availability

Samples of the compounds are available from the authors.
