# Supplementary material for: Sustainable Route for Synthesizing Aluminosilicate EU-1 Zeolite
Source: Molecules. 2021 Mar 8;26(5):1462. doi: 10.3390/molecules26051462 (PMC7962662; doi:10.3390/molecules26051462)
Supplement: Supplementary file 1 [file molecules-26-01462-s001.pdf]

Supporting Information

Sustainable Route for Synthesizing Aluminosilicate EU-1 Zeolite

Hao Xu [1], Jie Zhu [2], Xiong Wang [3], Chao Shen [1], Shengshen Meng [2], Kai Zheng [1], Chao Lei [1], and Longfeng Zhu [2,*]

[1] College of Biology and Environmental Engineering, Zhejiang Shuren University, Hangzhou 310015, P. R. China;
[2] College of Biological, Chemical Sciences and Engineering, Jiaxing University, Jiaxing 314001, P. R. China;
[3] Ningbo Rail Transit, Ningbo 315101, P. R. China.
* Correspondence: zhulf1988@mail.zjxu.edu.cn; Tel.: +86-573-8364-0131 (L.F.Z)

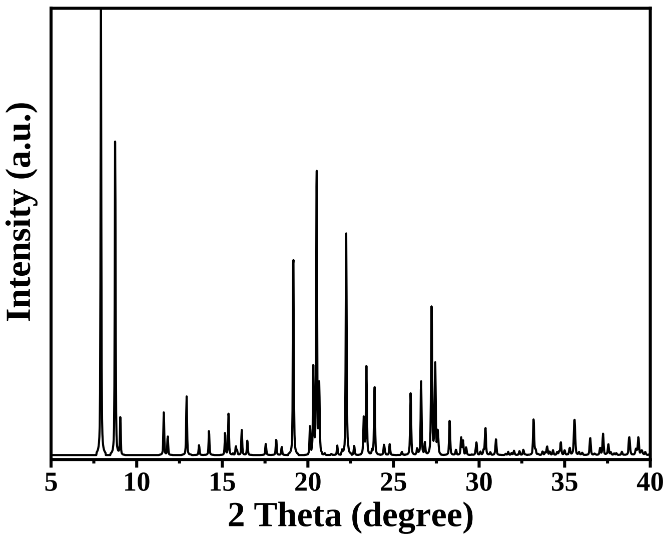

FigureS1. Simulated XRD pattern of the EU-1 zeolite.

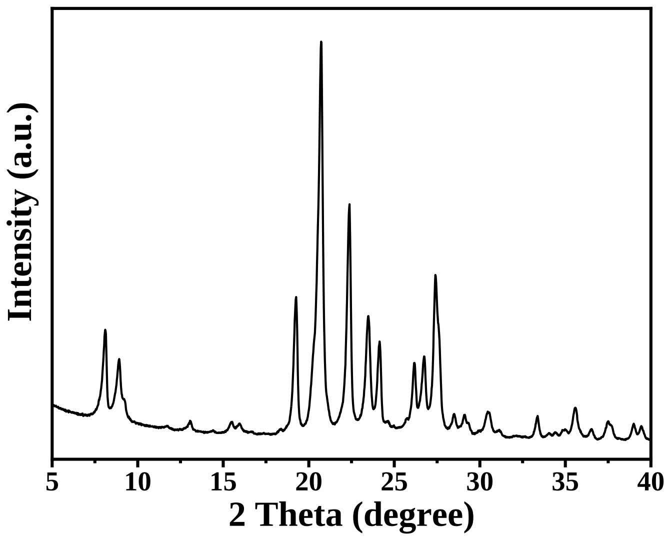

Figure S2. XRD pattern of the C-EU-1 zeolite.

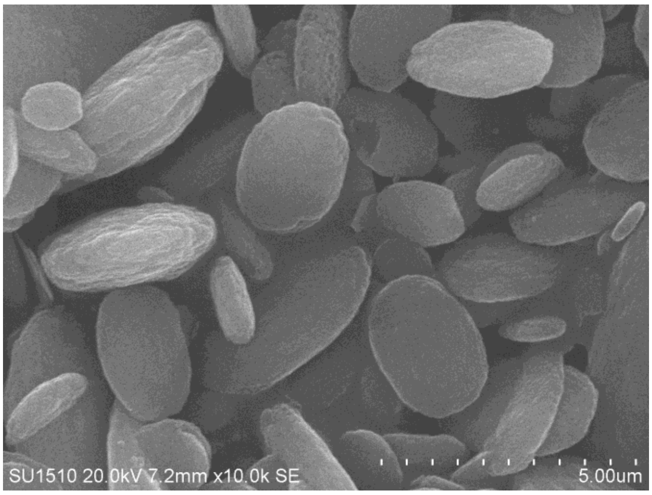

Figure S3. SEM image of the C-EU-1 zeolite.
